# Supplementary material for: Mitogenomic Characterization and Comparative Analysis of Three Egg Parasitoid Wasps Parasitizing Nilaparvata lugens (Stål)
Source: Insects. 2025 May 20;16(5):543. doi: 10.3390/insects16050543 (PMC12112357; doi:10.3390/insects16050543)
Supplement: Supplementary file 1 [file insects-16-00543-s001.zip › insects-3603381-supplementary.pdf]

Table S1. Primers used by sanger sequencing to obtain complete *P. nephotetticum* mitochondrial genome sequence.

	Primer	Sequence
1	C1-J1709	TTGTTATACCTGTTATAATAGGAG
2	C1-N2776	GATAATCTGAATATCGACGAG
3	C1-J2756	CAACATTTTTAGGTTTAAGAGG
4	C2-N3665	GAACATTGACCATAATATAAACC
5	C2-J3399	GTTTTAGGACATCAGTGATATTG
6	A6-N4552	ATGACCAGCAATTATATTTGC
7	A6-J4463	TTAGTTCCTCAAGGGACAC
8	C3-N5460	AACAAAATGTCAATACCAAAC
9	C3-J5470	CATCATTTTGGATTTGAAGC
10	N2-N993	TTAAAAATCCAAACATTGGCG
11	N2-J586	GGAATACCACCTTTTTATTATTG
12	N3-J5747	TCAGTTAATCGAATACCATTTTC
13	TG R	AAACCTATTAATTGGAAATTAATTATAC
14	TG F	TTAGTATAATTAATTTCCAATTAATAGG
15	TR R	TTTCAAGCCGAAATTGAAC
16	KF 2	TAATGTAATTTATATGTTTATATAGTTATAGT
17	SR-J14610	ATAATAGGGTATCTAATCCTAATTT
18	SR R	CTATTGCTGGCACTTAAAAT
19	SR-N14745	GCCAGCAATAGCGGT
20	LR-J13342	CTTTGTACAGTTAAAATACTGC
21	LR-N13000	TTACCTTAGGGATAACAGCGTAA
22	16SF	GGAATTAGACAAAAATTTTTATTCAC
23	16SR	CGCAAACTATTTTATCAATATGATC
24	N1-J12261	GATTTAGTTCAGCTAATAATCTAAC
25	N1-N12067	AATCGTACTCCTTTTGATTTAG
26	CB-J11335	CCAATTCATATTCAACCAGAAT
27	CB-N11526	GTGCTCCAATTCAAGTTAAAAT
28	CB-J10621	CAATTTGATGAAACTTTGGATC
29	CB-N10608	CCTAATAATGATCCAAAGTTTC
30	N4L-J9648	ACCTAAAACTCCTTCACTTAC
31	N4L-N9629	GTAAGTGAAGGAGTTTTAGG
32	N4-J8641	CCAGAAGAACAAATACCATG
33	N4-N8727	GTTGCTTATTCTTCTGTAGTAC
34	N5-J7572	AAGGTAACTGAGCACTTTT
35	N5-N7793	CCAAATATAATTAGAATTTTATTAGGT
36	N5-J7077	TTTGATCCTTAGAATAAAACCC
37	N5-N7211	GTTTATATGTTCAGGTATTATTATTC
38	C1-N1738	TTTATTCGAGGAAATGCTATATC
39	2-1 F	GTTTATTATTTGATAAAGGTTGAAGAG
40	2-1 R	TGTAATTGCTCCAGCTAAAAC
41	1093 F	GATACTTATTATGTAGTTGCTCATT
42	2-3 F	GTACCTGGTCGAATTAATCAAATT
43	2-3 R	CATTAGGTGTCCCTTGAGG
44	2-4 F	CTACTGGATTTCATGGTTTACATG
45	2-4 R	TATCAATAATAAAAAGGTGGTATTCC
46	2-5F	TGCTGGTTGATCTTCTAATAG
47	2-5 R	TTTAACTATACCCAAGTGTATGT

Table S2. Primers used by sanger sequencing to obtain *A. frequens* mitochondrial genome sequence.

	Primer	Sequence
1	C1-J1709	TTGTTATACCTGTAATAATAGGTG
2	C1-N2776	GATAATCAGAATATCGWCGNG
3	C1-J2756	CARCAYTTTTTAGGTTTAAGAG
4	C2-N3665	GAACATTGACCATAAAATAATC
5	C2-J3399	TCTATTGGTAATCAATGATATTG
6	A6-N4552	TTATATTWGCTGATAAACGAAC
7	C3-J5470	CATCATTTTGGATTTGAAGC
8	N5-N7211	TTTATATGTTCAGGAATTTTTATTC
9	N5-J7806	CCTAATAAAATTCTAATTAAATTTGG
10	N4-N8727	AACAAAATGATTTAAAATCATTAG
11	N4-J9172	ATATATATATACTAGCTTGAATTCG
12	CB-N10608	CCTAATAAWGAACCAAAATTTCA
13	CB-J10621	CWATTTGATGAAATTTTGGTTC
14	CB-N11526	GCWCCAATYCAWGTTAATA
15	CB-J11335	GTWCATATTCAACCAGAATG
16	N1-N12595	AGWRTWGCATTTTTAACTTTATTAG
17	N1-J12261	AAATTATTCTAACTTCATAWGAAATAG
18	LR-N13000	CTTTAGGGATAACAGCATAA
19	LR-J12888	CCGATTTAAACTCAAATCATG
20	SR-N14745	GCAATAGCGGTTATACAA
21	a1,129 J	GTTTTATCTATAGGTGCAGT
22	a3389 N	TATTCATARGATCARTATCAYTG
23	b4,071N	ACAAAATGTCAATATCAAGC
24	b2,196 J	TGTGGATTATTTTATGGTCA
25	c7,918N	GAACGAATTCAAGCTAGTAT
26	c7,094 J	TCCATGTTGTCTATATGAAA
27	d9,998N	CCTATAGGATTTGAAGAACC
28	d7,899J	ATACTAGCTTGAATTCGTTC
29	e16S-J	TTAATTCAACATCGAGGTC
30	e13,014 N	ATATATACAAATTGCCCGTC
31	f7793 N	TTAGGTTGRGATGGNYTAGG
32	f5715J	WTCYCCWTTYGAATGYGGAT
33	g7,918N	GAACGAATTCAAGCTAGTAT
34	g7,094J	TCCATGTTGTCTATATGAAA
35	hC3-N	TSWACAAAATGTCARTATCA
36	hC3-J-3033	TCTAWTATGGCAGATTAGTGTA

Table S3. Mitogenomes of Chalcidoidea and outgroups used in this study.

Family	Species	Accession number	Length(bp)
Mymaridae	*Gonatocerus* sp.	MF776883	15,554
	Anagrus frequens	PV449103	15,510
	Anagrus nilaparvatae	PV449104	15,429
Agaonidae	*Dolichoris vasculosae*	MT947596	15,334
	Eupristina koningsbergeri	MT947597	15,400
	Kradibia gibbosae	MT947598	15,281
	Wiebesia pumilae	MT947601	15,090
Aphelinidae	*Encarsia obtusiclava*	MG813798	14,197
	Encarsia formosa	MG813797	17,736
Chalcididae	*Brachymeria lasus*	MZ615567	15,147
	Brachymeria sp.	MG923487	15,092
	Haltichella nipponensis	MZ615568	15,334
Eulophidae	*Chouioia cumea*	NC_060368	14,930
	Tamarixia radiata	MN123622	14,752
	Necremnus tutae	NC_053857	15,252
Eurytomidae	*Eurytoma* sp.	MG923494	17,267
	Sycophila sp.	MT947603	14,665
Pteromalidae	*Pteromalus puparum*	MH051556	18,217
	Philotrypesis tridentata	MT947602	14,747
	Philotrypesis pilosa	JF808723	15,122
	Pachyneuron aphidis	MK577639	15,137
	Nasonia oneida	MT762278	14,811
	Nasonia vitripennis	MT712141	14,791
	Nasonia longicornis	MT755966	14,925
	Nasonia giraulti	MT762280	15,291
	Anisopteromalus calandrae	MW817149	15,954
Encyrtidae	*Encyrtus aurantii*	OR120384	15,397
	Encyrtus rhodococcusiae	NC_051460	15,694
	Encyrtus infelix	MH729198	15698
	Encyrtus sasakii	MK111647	15,708
	Encyrtus eulecaniumiae	MK189128	15,692
	Aenasius arizonensis	MK630013	15373
	Diaphorencyrtus aligarhensis	MN274569	16,264
	Platencyrtus parkeri	MN296710	13,393
	Metaphycus eriococci	MW255970	15,749
Trichogrammatidae	*Trichogramma cacaeciae*	NC_069040	16,034
	Trichogramma dendrolimi	KU836507	16,878
	Trichogramma pretiosum	NC_069039	16,227
	Trichogramma ostriniae	NC_039535	16,472
	Trichogramma chilonis	MW789210	16,147
	Trichogramma japonicum	NC_039534	15,962
	Megaphragma amalphitanum	NC_028196	15,041
	Trichogramma kaykai	PQ667799	16,399
	Pseudoligosita yasumatsui	NC_082212	16,036
	Pseudoligosita nephotteticum	PV449102	15,889
Torymidae	*Torymus* sp.	MG923516	16,826
Cynipoidea	*Trichagalma acutissimae*	MN928529	16,078
Ibaliidae	*lbalia leucospoides*	KJ814197	17,212

Table S4. Characteristics of the mitochondrial genome of *P. nephotetticum*.

Features	Strand	Start sites	Stop sites	Size (bp)	Start condon	Stop condon	Intergenic nucleotides (bp)
cox1	+	1	1,536	1,536	ATG	TAA	6
trnL2(taa)	+	1,543	1,608	66			0
cox2	+	1,609	2,286	678	ATT	TAA	5
trnD(gtc)	+	2,292	2,360	69			143
trnK(ttt)	–	2,504	2,572	69			50
atp8	+	2,623	2,784	162	ATT	TAA	-7
atp6	+	2,778	3,449	672	ATG	TAA	-1
cox3	+	3,449	4,234	786	ATG	TAA	23
trnC(gca)	+	4,258	4,324	67			34
trnI(gat)	+	4,359	4,427	69			46
nad2	+	4,474	5,463	990	ATT	TAA	2
trnW(tca)	+	5,466	5,536	71			7
trnY(gta)	–	5,544	5,611	68			2
trnS1(tct)	–	5,614	5,674	61			0
trnN(gtt)	–	5,675	5,742	68			-3
trnM(cat)	+	5,740	5,806	67			2
trnG(tcc)	+	5,809	5,875	67			0
AT-rich region		5,876	6,341	466			0
trnV(tac)	+	6,342	6,408	67			-9
rrnS	+	6,400	7,164	765			6
nad3	+	7,171	7,521	351	ATT	TAA	39
trnQ(ttg)	–	7,557	7,626	70			6
trnR(tcg)	+	7,633	7,697	65			11
trnA(tgc)	+	7,709	7,773	65			-34
rrnL	+	7,740	9,105	1,366			-21
trnL1(tag)	+	9,085	9,152	68			0
nad1	+	9,153	10,091	939	ATA	TAA	-10
trnS2(tga)	–	10,082	10,145	64			13
cob	–	10,159	11,301	1,143	ATG	TAA	7
nad6	–	11,309	11,809	501	ATT	TAA	95
trnP(tgg)	+	11,905	11,968	64			4
trnT(tgt)	–	11,973	12,035	63			18
nad4l	+	12,054	12,344	291	ATA	TAA	-7
nad4	+	12,338	13,684	1,347	ATG	TAA	-1
trnH(gtg)	+	13,684	13,754	71			306
nad5	+	14,061	15,770	1,710	ATA	TAA	-27
trnF(gaa)	+	15,751	15,815	65			2
trnE(ttc)	–	15,818	15,884	67			3

Table S5. Characteristics of the mitochondrial genome of *A. frequens.*

Features	Strand	Start sites	Stop sites	Size (bp)	Start condon	Stop condon	Intergenic nucleotides (bp)
cox1	+	1	1,536	1,536	ATT	TAA	42
trnL2(taa)	+	1,579	1,646	68			0
cox2	+	1,647	2,312	666	ATA	TAA	12
trnK(ttt)	+	2,325	2,391	67			17
trnD(gtc)	+	2,409	2,476	68			9
atp8	+	2,486	2,636	151	ATA	T	7
atp6	+	2,644	3,324	681	ATT	TAA	6
cox3	+	3,331	4,116	786	ATG	TAA	36
trnG(tcc)	+	4,153	4,225	73			-4
nad3	+	4,222	4,575	354	ATA	TAA	17
trnR(tcg)	+	4,593	4,649	57			-1
trnN(gtt)	+	4,649	4,713	65			-1
trnS1(tct)	+	4,713	4,774	62			5
trnF(gaa)	+	4,780	4,849	70			3
trnM(cat)	-	4,853	4,921	69			78
trnQ(ttg)	+	5,000	5,071	72			65
trnE(ttc)	-	5,137	5,204	68			3
nad5	-	5,208	6,873	1,666	ATT	T	3
trnH(gtg)	-	6,877	6,947	71			0
nad4	-	6,948	8,270	1,323	ATA	TAA	-4
nad4l	-	8,267	8,524	258	ATA	TAA	34
trnT(tgt)	+	8,559	8,623	65			5
trnP(tgg)	-	8,629	8,699	71			3
nad6	+	8,703	9,275	573	ATG	TAG	3
cob	+	9,279	10,421	1,143	ATG	TAA	4
trnS2(tga)	+	10,426	10,493	68			7
nad1	-	10,501	11,454	954	ATA	TAA	84
rrnL	-	11,539	12,720	1,182			0
trnA(tgc)	-	12,721	12,787	67			0
rrnS	-	12,788	13,569	782			112
AT-rich region	+	13,682	14,076	395			-1
trnV(tac)	+	14,076	14,141	66			-3
trnI(atc)	-	14,139	14,204	66			2
nad2	+	14,207	15,244	1,038	ATA	TAA	-3
trnW(tca)	+	15,242	15,311	70			5
trnC(gca)	-	15,317	15,391	75			3
trnY(gaa)	-	15,395	15,461	67			0

Table S6. Characteristics of the mitochondrial genome of *A. nilaparvatae*

Features	Strand	Start sites	Stop sites	Size (bp)	Start condon	Stop condon	Intergenic nucleotides (bp)
cox1	+	1	1,536	1,536	ATT	TAA	8
trnL2(taa)	+	1,545	1,611	67			0
cox2	+	1,612	2,274	663	ATT	TAA	7
trnK(ttt)	+	2,282	2,348	67			0
trnD(gtc)	+	2,349	2,415	67			9
atp8	+	2,425	2,575	151	ATA	T	10
atp6	+	2,586	3,266	681	ATT	TAG	1
cox3	+	3,268	4,053	786	ATG	TAA	22
trnG(tcc)	+	4,076	4,139	64			-3
nad3	+	4,137	4,490	354	ATA	TAA	1
trnR(tcg)	+	4,492	4,546	55			-2
trnN(gtt)	+	4,545	4,611	67			-3
trnS1(tct)	+	4,609	4,670	62			9
trnF(gaa)	+	4,680	4,744	65			2
trnM(cat)	-	4,747	4,813	67			23
trnQ(ttg)	+	4,837	4,903	67			30
trnE(ttc)	-	4,934	5,000	67			2
nad5	-	5,003	6,676	1,674	ATT	TAA	3
trnH(gtg)	-	6,680	6,747	68			0
nad4	-	6,748	8,070	1,323	ATA	TAA	-4
nad4l	-	8,067	8,324	258	ATA	TAA	85
trnT(tgt)	+	8,410	8,475	66			1
trnP(tgg)	-	8,477	8,542	66			1
nad6	+	8,544	9,114	571	ATG	T	0
cob	+	9,115	10,251	1,137	ATG	TAA	4
trnS2(tga)	+	10,256	10,322	67			7
nad1	-	10,330	11,283	954	ATA	TAA	32
rrnL	-	11,316	12,547	1,232			0
trnA(tgc)	-	12,548	12,617	70			-1
rrnS	-	12,617	13,437	821			0
AT-rich region	+	13,438	14,039	602			1
trnV(tac)	+	14,041	14,105	65			0
trnI(atc)	-	14,106	14,173	68			19
nad2	+	14,193	15,233	1,041	ATA	TAA	-1
trnW(tca)	+	15,233	15,297	65			-1
trnC(gca)	-	15,297	15,363	67			0
trnY(gaa)	-	15,364	15,429	66			0

Table S7. Relative synonymous codon usage (RSCU) values of three egg parasitoid wasps.

Specie	AA	Condon	Count	RSCU	AA	Condon	Count	RSCU	AA	Condon	Count	RSCU	AA	Condon	Count	RSCU
P. nephotetticum	Phe	UUU(F)	424	1.74	Ser2	UCU(S)	46	1.3	Tyr	UAU(Y)	290	1.82	Cys	UGU(C）	38	1.52
		UUC(F)	62	0.26		UCC(S)	19	0.54		UAC(Y)	29	0.18		UGC(C）	12	0.48
	Leu1	UUA(L)	402	4.68		UCA(S)	83	2.34		UAA(*)	176	1.64	Trp	UGA(W)	59	1.28
		UUG(L)	44	0.51		UCG(S)	13	0.37		UAG(*)	39	0.36		UGG(W)	33	0.72
	Leu2	CUU(L)	34	0.4	Pro	CCU(P)	29	2.47	His	CAU(H)	36	1.89	Arg	CGU(R)	10	1.74
		CUC(L)	6	0.07		CCC(P)	1	0.09		CAC(H)	2	0.11		CGC(R)	0	0
		CUA(L)	20	0.23		CCA(P)	16	1.36	Gln	CAA(Q)	22	1.42		CGA(R)	13	2.26
		CUG(L)	9	0.1		CCG(P)	1	0.09		CAG(Q)	9	0.58		CGG(R)	0	0
	Ile	AUU(I)	403	1.91	Thr	ACU(T)	23	1.31	Asn	AAU(N)	277	1.87	Ser1	AGU(S)	35	0.99
		AUC(I)	20	0.09		ACC(T)	11	0.63		AAC(N)	19	0.13		AGC(S)	8	0.23
	Met	AUA(M)	298	1.81		ACA(T)	35	2	Lys	AAA(K)	135	1.71		AGA(S)	51	1.44
		AUG(M)	31	0.19		ACG(T)	1	0.06		AAG(K)	23	0.29		AGG(S)	29	0.82
	Val	GUU(V)	55	2	Ala	GCU(A)	15	1.88	Asp	GAU(D)	45	1.8	Gly	GGU(G)	35	1.67
		GUC(V)	5	0.18		GCC(A)	0	0		GAC(D)	5	0.2		GGC(G)	0	0
		GUA(V)	42	1.53		GCA(A)	17	2.13	Glu	GAA(E)	59	1.76		GGA(G)	44	2.1
		GUG(V)	8	0.29		GCG(A)	0	0		GAG(E)	8	0.24		GGG(G)	5	0.24
A. frequens	Phe	UUU(F)	415	1.77	Ser2	UCU(S)	67	2.31	Tyr	UAU(Y)	303	1.81	Cys	UGU(C）	33	1.61
		UUC(F)	55	0.23		UCC(S)	8	0.28		UAC(Y)	31	0.19		UGC(C）	8	0.39
	Leu1	UUA(L)	374	4.89		UCA(S)	50	1.72		UAA(*)	192	2.02	Trp	UGA(W)	56	0.59
		UUG(L)	41	0.54		UCG(S)	10	0.34		UAG(*)	37	0.39		UGG(W)	27	1
	Leu2	CUU(L)	20	0.26	Pro	CCU(P)	35	2.12	His	CAU(H)	47	1.88	Arg	CGU(R)	11	0.72
		CUC(L)	4	0.05		CCC(P)	1	0.06		CAC(H)	3	0.12		CGC(R)	0	0
		CUA(L)	15	0.2		CCA(P)	28	1.7	Gln	CAA(Q)	42	1.95		CGA(R)	12	0.78
		CUG(L)	5	0.07		CCG(P)	2	0.12		CAG(Q)	1	0.05		CGG(R)	3	0.2
	Ile	AUU(I)	442	1.83	Thr	ACU(T)	32	1.51	Asn	AAU(N)	294	1.87	Ser1	AGU(S)	28	0.97
		AUC(I)	29	0.12		ACC(T)	14	0.66		AAC(N)	21	0.13		AGC(S)	11	0.38
	Met	AUA(M)	254	1.05		ACA(T)	34	1.6	Lys	AAA(K)	151	1.74		AGA(S)	50	3.26
		AUG(M)	32	1		ACG(T)	5	0.24		AAG(K)	23	0.26		AGG(S)	16	1.04
	Val	GUU(V)	45	1.89	Ala	GCU(A)	20	2.42	Asp	GAU(D)	56	1.93	Gly	GGU(G)	44	1.85
		GUC(V)	1	0.04		GCC(A)	0	0		GAC(D)	2	0.07		GGC(G)	2	0.08
		GUA(V)	44	1.85		GCA(A)	13	1.58	Glu	GAA(E)	47	1.68		GGA(G)	42	1.77
		GUG(V)	5	0.21		GCG(A)	0	0		GAG(E)	9	0.32		GGG(G)	7	0.29
A. nilaparvatae	Phe	UUU(F)	440	1.75	Ser2	UCU(S)	28	0.86	Tyr	UAU(Y)	308	1.84	Cys	UGU(C）	37	1.51
		UUC(F)	62	0.25		UCC(S)	18	0.55		UAC(Y)	26	0.16		UGC(C）	12	0.49

Leu1	UUA(L)	404	5.04		UCA(S)	96	2.95		UAA(*)	131	1.76	Trp	UGA(W)	65	0.87
	UUG(L)	38	0.47		UCG(S)	10	0.31		UAG(*)	27	0.36		UGG(W)	22	1
Leu2	CUU(L)	18	0.22	Pro	CCU(P)	20	1.7	His	CAU(H)	35	1.75	Arg	CGU(R)	4	0.22
	CUC(L)	4	0.05		CCC(P)	3	0.26		CAC(H)	5	0.25		CGC(R)	0	0
	CUA(L)	10	0.12		CCA(P)	22	1.87	Gln	CAA(Q)	34	1.55		CGA(R)	12	0.67
	CUG(L)	7	0.09		CCG(P)	2	0.17		CAG(Q)	10	0.45		CGG(R)	1	0.06
Ile	AUU(I)	382	1.77	Thr	ACU(T)	33	1.39	Asn	AAU(N)	328	1.86	Ser1	AGU(S)	35	1.08
	AUC(I)	33	0.15		ACC(T)	19	0.8		AAC(N)	25	0.14		AGC(S)	8	0.25
Met	AUA(M)	233	1.08		ACA(T)	37	1.56	Lys	AAA(K)	165	1.69		AGA(S)	63	3.53
	AUG(M)	41	1		ACG(T)	6	0.25		AAG(K)	30	0.31		AGG(S)	27	1.51
Val	GUU(V)	56	2.31	Ala	GCU(A)	20	1.78	Asp	GAU(D)	45	1.7	Gly	GGU(G)	41	1.78
	GUC(V)	4	0.16		GCC(A)	1	0.09		GAC(D)	8	0.3		GGC(G)	2	0.09
	GUA(V)	32	1.32		GCA(A)	20	1.78	Glu	GAA(E)	38	1.65		GGA(G)	42	1.83
	GUG(V)	5	0.21		GCG(A)	4	0.36		GAG(E)	8	0.35		GGG(G)	7	0.3

Table S8. Ka/Ka value in protein-coding genes of mitochondrial genomes of 13 species in Mymaridae and Trichogrammatidae.

Species \ Genes		atp6	atp8	cox1	cox2	cox3	cob	nad1	nad2	nad3	nad4	nad4L	nad5	nad6
ka	*A. frequens*	0.38	0.93	0.26	0.37	0.41	0.30	0.41	0.70	0.47	0.50	0.61	0.53	0.65
	A. nilaparvatae	0.39	1.05	0.26	0.38	0.47	0.30	0.38	0.73	0.48	0.46	0.62	0.52	0.66
	Gonatocerus sp.	0.46	0.90	0.25	0.40	0.41	0.33	0.42	0.77	0.45	0.50	0.76	0.52	0.83
	M.amalphitanum	0.39	0.84	0.23	0.38	0.40	0.29	0.36	0.72	0.50	0.49	0.63	0.47	0.88
	P. nephotteticum	0.43	0.77	0.23	0.42	0.42	0.30	0.43	0.85	0.76	0.52	0.65	0.47	0.86
	P. yasumatsui	0.41	0.83	0.23	0.40	0.42	0.31	0.43	0.69	0.43	0.52	0.63	0.46	0.88
	T. cacaeciae	c	1.06	0.23	0.39	0.42	0.33	0.37	0.68	0.39	0.50	0.63	0.48	0.87
	T. chilonis	0.42	0.86	0.22	0.39	0.41	0.33	0.35	0.68	0.41	0.48	0.64	0.49	0.83
	T. dendrolimi	0.40	1.12	0.22	0.39	0.39	0.33	0.34	0.70	0.43	0.48	0.64	0.48	0.85
	T. japonicum	0.41	0.92	0.22	0.40	0.41	0.31	0.36	0.69	0.44	0.46	0.67	0.50	0.82
	T. kaykai	0.42	0.96	0.22	0.39	0.42	0.34	0.37	0.69	0.40	0.48	0.66	0.48	0.87
	T. ostriniae	0.42	1.04	0.22	0.38	0.41	0.34	0.37	0.69	0.42	0.50	0.66	0.48	0.84
	T. pretiosum	0.41	0.99	0.23	0.39	0.42	0.34	0.36	0.68	0.40	0.48	0.67	0.49	0.84
ks	*A. frequens*	0.40	0.34	0.57	0.45	0.58	0.65	0.39	0.39	0.42	0.36	0.29	0.52	0.29
	A. nilaparvatae	0.38	0.30	0.66	0.54	0.53	0.60	0.46	0.33	0.48	0.43	0.44	0.53	0.32
	Gonatocerus sp.	0.43	0.31	0.46	0.40	0.44	0.55	0.36	0.34	0.22	0.31	0.35	0.34	0.27
	M.amalphitanum	0.48	0.36	0.58	0.52	0.43	0.58	0.38	0.44	0.47	0.41	0.40	0.39	0.31
	P. nephotteticum	0.45	0.29	0.53	0.47	0.48	0.61	0.43	0.49	0.45	0.38	0.32	0.41	0.22
	P. yasumatsui	0.55	0.33	0.56	0.53	0.49	0.56	0.45	0.38	0.44	0.40	0.23	0.40	0.30
	T. cacaeciae	0.56	0.27	0.56	0.46	0.47	0.54	0.38	0.40	0.66	0.49	0.39	0.46	0.24
	T. chilonis	0.49	0.39	0.62	0.44	0.52	0.57	0.41	0.41	0.60	0.47	0.40	0.44	0.33
	T. dendrolimi	0.60	0.48	0.67	0.43	0.57	0.60	0.38	0.53	0.58	0.47	0.55	0.43	0.36
	T. japonicum	0.53	0.28	0.64	0.58	0.61	0.69	0.43	0.45	0.49	0.39	0.37	0.38	0.27
	T. kaykai	0.47	0.31	0.63	0.53	0.63	0.61	0.32	0.45	0.50	0.46	0.47	0.38	0.30
	T. ostriniae	0.46	0.41	0.61	0.46	0.52	0.57	0.42	0.37	0.52	0.45	0.46	0.44	0.27
	T. pretiosum	0.48	0.31	0.71	0.53	0.53	0.53	0.40	0.41	0.54	0.41	0.39	0.47	0.29
ka/ks	*A. frequens*	0.97	2.73	0.45	0.83	0.70	0.46	1.04	1.78	1.11	1.38	2.13	1.02	2.23
	A. nilaparvatae	1.03	3.54	0.39	0.69	0.90	0.50	0.83	2.19	1.00	1.06	1.41	0.97	2.03
	Gonatocerus sp.	1.08	2.89	0.53	0.99	0.93	0.61	1.17	2.24	2.07	1.64	2.15	1.53	3.04
	M.amalphitanum	0.82	2.31	0.39	0.73	0.92	0.49	0.96	1.65	1.08	1.19	1.57	1.20	2.83
	P. nephotteticum	0.96	2.71	0.43	0.91	0.88	0.49	1.00	1.73	1.70	1.37	2.02	1.15	3.83
	P. yasumatsui	0.74	2.54	0.41	0.76	0.86	0.55	0.95	1.79	0.97	1.31	2.70	1.16	2.97
	T. cacaeciae	0.77	3.92	0.40	0.84	0.90	0.60	0.97	1.73	0.59	1.03	1.61	1.03	3.65
	T. chilonis	0.86	2.19	0.36	0.88	0.79	0.58	0.86	1.66	0.69	1.04	1.60	1.11	2.55
	T. dendrolimi	0.66	2.35	0.33	0.91	0.68	0.55	0.91	1.32	0.75	1.02	1.17	1.10	2.37
	T. japonicum	0.79	3.29	0.35	0.69	0.68	0.45	0.83	1.55	0.89	1.18	1.81	1.30	3.06
	T. kaykai	0.89	3.06	0.36	0.74	0.66	0.55	1.14	1.54	0.81	1.06	1.40	1.28	2.86
	T. ostriniae	0.92	2.53	0.37	0.84	0.79	0.59	0.86	1.88	0.81	1.10	1.43	1.10	3.10
	T. pretiosum	0.86	3.16	0.32	0.74	0.79	0.64	0.90	1.66	0.73	1.16	1.69	1.05	2.92

Table S9. The best fit DNA model of each locus in datasets PCG123 and PCG12

PCG123 dataset			PCG12 dataset		
	Best fit DNA model			Best fit DNA model	
Locus	ML	BI	Locus	ML	BI
atp6-1condon	GTR+F+R6	GTR+G+I	12atp6-1	GTR+F+R6	GTR+G+I
atp6-2condon		GTR+G+I	12atp6-2		GTR+G
atp6-3condon		GTR+G			
atp8-1condon		HKY+G	12atp8-1		GTR+G
atp8-2condon		HKY+G	12atp8-2		GTR+G
atp8-3condon		GTR+G			
cox1-1condon		GTR+G+I	12cox1-1		GTR+G+I
cox1-2condon		GTR+G	12cox1-2		GTR+G
cox1-3condon		GTR+G+I			
cox2-1condon		GTR+G	12cox2-1		HKY+G
cox2-2condon		GTR+G	12cox2-2		HKY+G
cox2-3condon		GTR+G+I			
cox3-1condon		GTR+G+I	12cox3-1		GTR+G+I
cox3-2condon		GTR+G	12cox3-2		GTR+G+I
cox3-3condon		GTR+G			
cytb-1condon		GTR+G	12cytb-1		GTR+G
cytb-2condon		GTR+G+I	12cytb-2		GTR+G+I
cytb-3condon		GTR+G			
nad1-1condon		GTR+G+I	12nad1-1		GTR+G+I
nad1-2condon		GTR+G+I	12nad1-2		GTR+G+I
nad1-3condon		GTR+G			
nad2-1condon		GTR+G+I	12nad2-1		GTR+G+I
nad2-2condon		GTR+G+I	12nad2-2		GTR+G+I
nad2-3condon		HKY+G+I			
nad3-1condon		GTR+G+I	12nad3-1		GTR+G+I
nad3-2condon		HKY+G	12nad3-2		HKY+G
nad3-3condon		HKY+G			
nad4-1condon		GTR+G+I	12nad4-1		GTR+G+I
nad4-2condon		GTR+G	12nad4-2		GTR+G
nad4-3condon		GTR+G+I			
nd4L-1condon		GTR+G	12nd4L-1		GTR+G
nd4L-2condon		GTR+G	12nd4L-2		GTR+G
nd4L-3condon		HKY+G			
nad4-1condon		GTR+G+I	12nad4-1		GTR+G+I
nad4-2condon		GTR+G+I	12nad4-2		GTR+G+I
nad4-3condon		GTR+G			
nad5-1condon		GTR+G+I	12nad5-1		GTR+G+I
nad5-2condon		GTR+G+I	12nad5-2		GTR+G+I
nad5-3condon		GTR+G			
nad6-1condon		GTR+G			
nad6-2condon		HKY+G+I	12nad6-1		GTR+G
nad6-3condon		HKY+G+I	12nad6-2		HKY+G+I

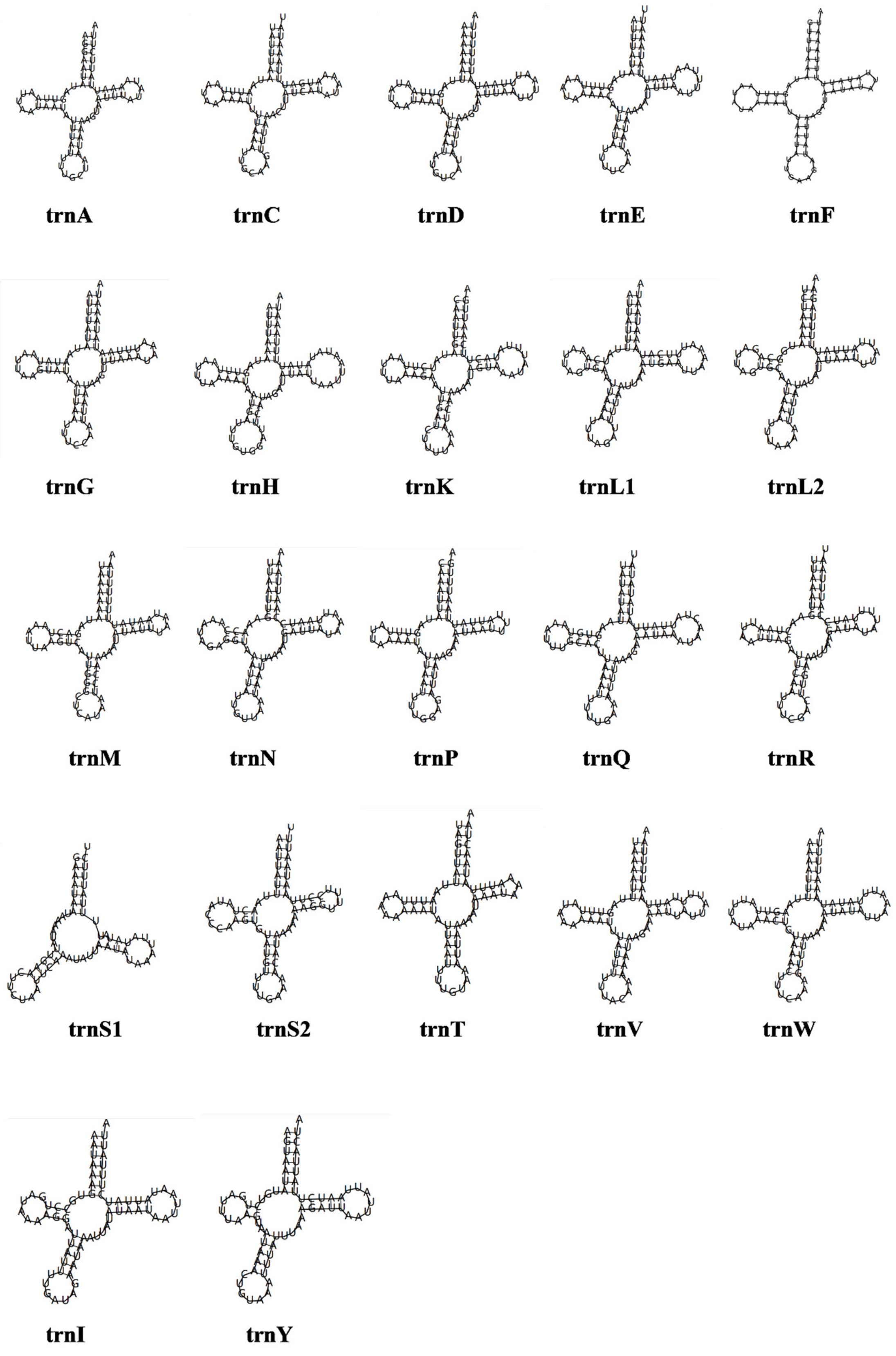

Figure S1. Predicted secondary structures of the 22 tRNA genes in *P. nephotetticum* mitogenome.

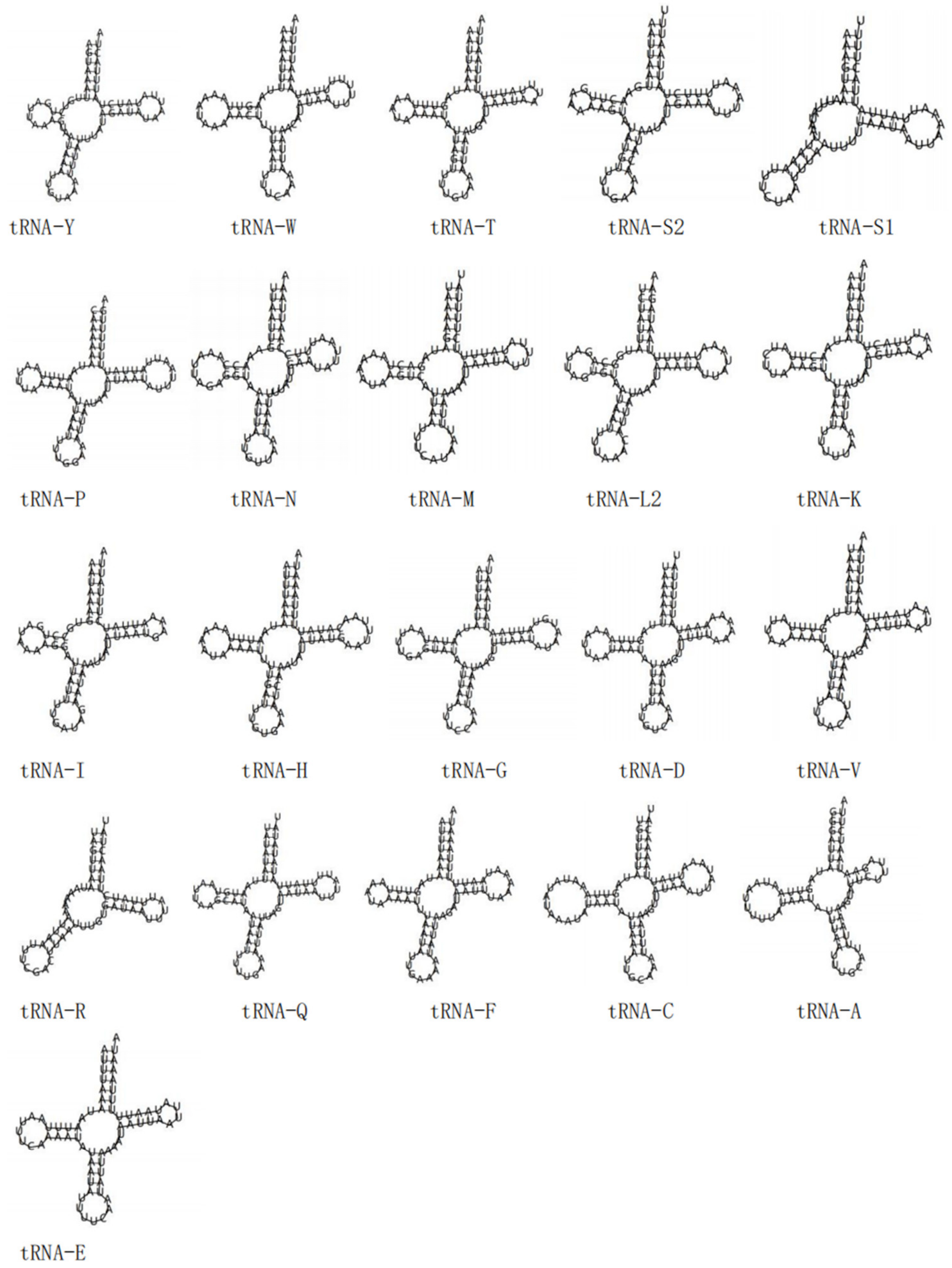

Figure S2. Predicted secondary structures of the 21 tRNA genes in *A. frequens* mitogenome.

稻

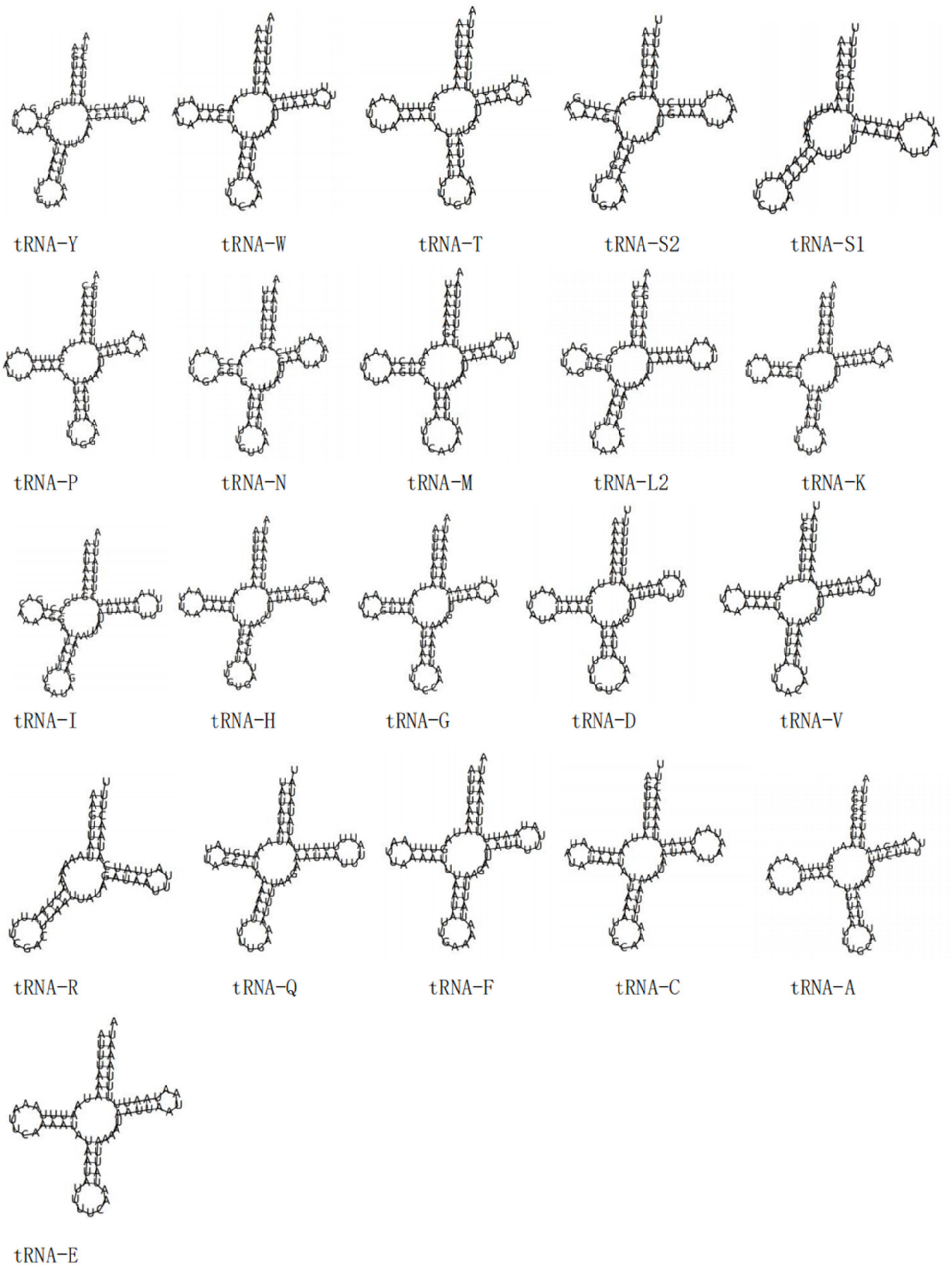

Figure S3. Predicted secondary structures of the 21 tRNA genes in *A. nilaparvatae* mitogenome.

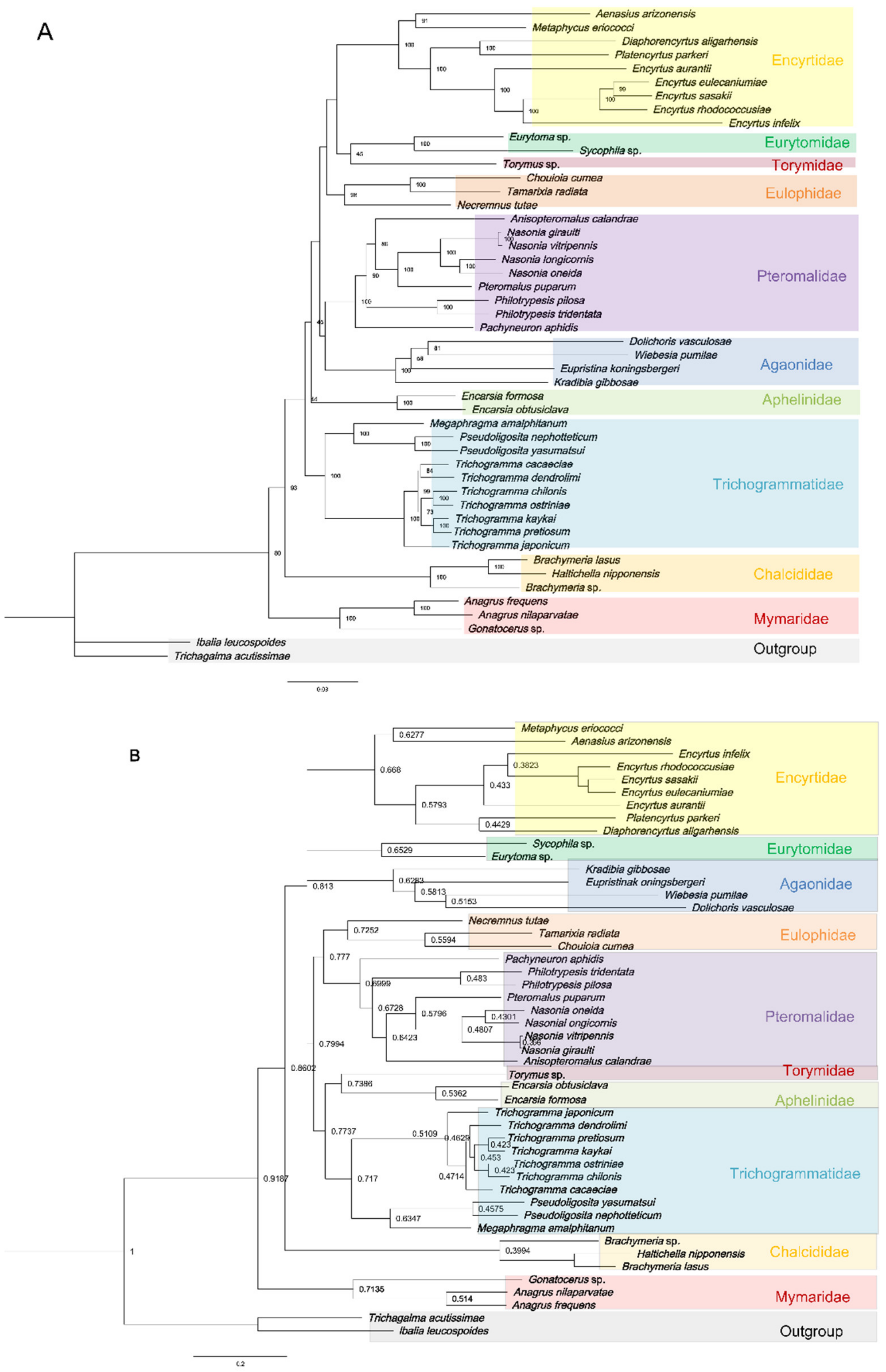

Figure S4. Phylogenetic trees of Pentatomidae inferred by ML (A) and BI (B) analysis based on dataset PCG12R.

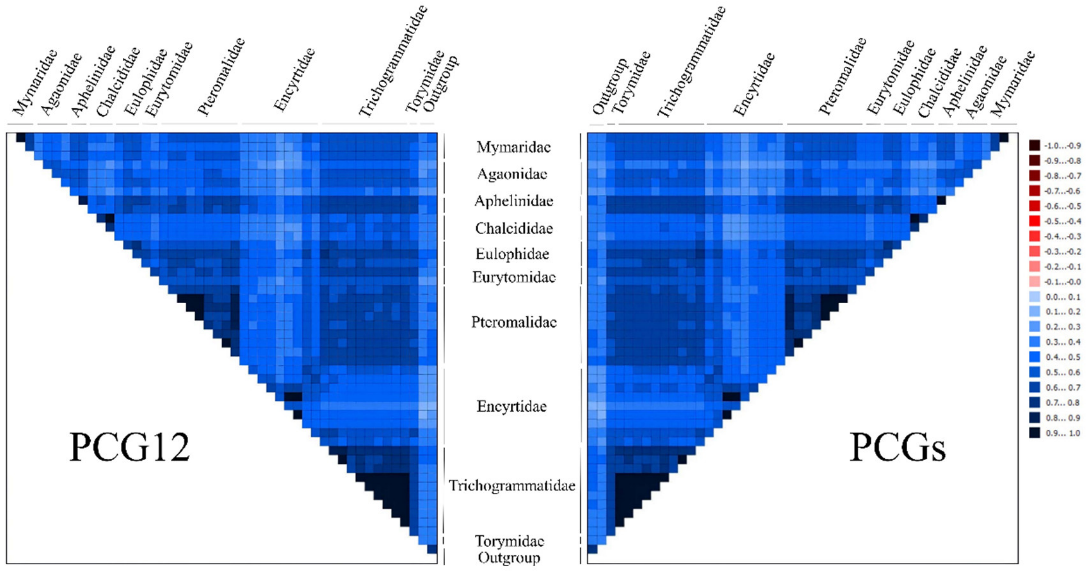

Figure S5. Compositional heterogeneity analysis in PCG12 and PCGs.
